# Lifestyle exercise attenuates immunosenescence; flow cytometry analysis

**DOI:** 10.1186/s12877-021-02128-7

**Published:** 2021-03-22

**Authors:** Anna Tylutka, Barbara Morawin, Artur Gramacki, Agnieszka Zembron-Lacny

**Affiliations:** 1 Department of Applied and Clinical Physiology, Collegium Medicum University of Zielona Gora, 28 Zyty Str., 65-046 Zielona Gora, Poland; 2 Faculty of Computer, Electrical and Control Engineering, Institute of Control and Computation Engineering University of Zielona Gora, Zielona Gora, Poland

**Keywords:** Lymphocytes T, Obesity, Physical activity, Successful ageing

## Abstract

**Background:**

Interaction of physical activity and overall immune profile is very complex and depends on the intensity, duration and frequency of undertaken physical activity, the exposure to cytomegalovirus (CMV) infection and the age-related changes in the immune system. Daily physical activity, which particularly influences immunity, declines dramatically with age. Therefore, the aim of the study was to explain whether physical activity sustained throughout life can attenuate or reverse immunosenescence.

**Methods:**

Ninety-nine older adults (60–90 years) were recruited for the study. According to the 6-min walk test (6WMT), the Åstrand-Ryhming bike test (VO_2_max) and Community Healthy Activities Model Program for Seniors (CHAMPS) questionnaire, the individuals were classified as physically active (*n* = 34) and inactive (*n* = 20) groups. The analysis of T lymphocytes between active vs. inactive participants was performed using eight-parameter flow cytometry.

**Results:**

Analysis of the baseline peripheral naïve and memory T lymphocytes showed a significant relationship of lifestyle exercise with the CD4/CD8 ratio. Above 50% of physically active participants demonstrated the CD4/CD8 ratio ≥ 1 or ≤ 2.5 contrary to the inactive group who showed the ratio < 1. The older adults with the result of 6WMT > 1.3 m/s and VO_2_max > 35 mL/kg/min had a significantly higher CD4^+^CD45RA^+^ T lymphocyte percentage and also a higher ratio of CD4^+^CD45RA^+^/CD4^+^CD45RO^+^. Interestingly, in active older adults with IgG CMV^+^ (*n* = 30) the count of CD4^+^CD45RA^+^ T lymphocytes was higher than in the inactive group with IgG CMV^+^ (*n* = 20).

**Conclusion:**

Based on the flow cytometry analysis, we concluded that lifestyle exercise could lead to rejuvenation of the immune system by increasing the percentage of naïve T lymphocytes or by reducing the tendency of the inverse CD4/CD8 ratio.

## Background

Immune function deregulates with age in the process commonly called immunosenescence. The characteristics of immunosenescence are related to a weaker response to new infections and vaccination as well as diminished anti-tumor immunosurveillance with increased self-reactivity and chronic systemic inflammation. Age-related thymus involution is a dynamic process that influences overall T lymphocytes development [1]. The T lymphocyte pool is part of subpopulations of antigen-inexperienced naïve T lymphocytes and antigen-experienced memory T lymphocytes. The human immune compartment is composed of ~ 10^12^ T lymphocytes in total, ~ 10^11^ of which are naïve [2]. During the process of ageing, the population of naïve T lymphocytes decreases, while the population of memory T lymphocytes undergoes intensive proliferation [3–5]. An increase in memory T lymphocytes reinforces immunological memory of previously encountered antigens, thereby augmenting the existent immune protection. The remaining naïve T lymphocyte pool experiences a loss of T lymphocyte receptor (TCR) ‘structural diversity’ [6, 7]. The appropriate range of antigen specificity is ensured by the diversity of T lymphocyte clones associated with a different number of TCR complexes [2]. Ageing affects naïve CD4^+^ T and CD8^+^ T lymphocyte counts in a different way. The number of CD4^+^naïve T lymphocytes is stable for most of the lifespan but it markedly declines around the age of 70 years. Contrastingly, CD8^+^ naïve T lymphocytes appear to be more susceptible to apoptosis and therefore are more sensitive to age-related changes. Homeostatic proliferation plays an important role in the maintenance of the number of naïve T lymphocytes, and cytokine IL-7 particularly supports the survival of CD8^+^naïve T lymphocytes [8]. The immunosenescence-related disproportion in T lymphocytes increases a risk of infectious diseases and contributes to the cardiovascular, metabolic, autoimmune, and neurodegenerative diseases [9].

As ageing is a natural process, the risk associated with relatively invasive surgical and immune-therapeutic procedures, i.e. gene and/or cytokine therapy and monoclonal antibody therapy, in otherwise healthy people is unlikely to be deemed acceptable. Of late, there has been some interest in the changes to modifiable factors, such as physical activity and certain lifestyle factors, which can significantly attenuate immunosenescence [10, 11]. In their latest study, Wong et al. [12] revealed that an immune response to the vaccination was more pronounced in physically active older adults females. However, the majority of studies concentrate on the effects of long-term physical activity on immunity in elite athletes, immunity in active and inactive young participants or they only document the immune changes following an exercise training intervention or physical therapy [10, 13]. Daily physical activity which particularly affects immunity and dramatically declines with age has not been widely investigated yet.

The interaction of lifestyle exercise and immunity is very complex and yet to be clarified. Regular physical activity including cardiovascular and resistance exercise has been associated with lower levels of pro-inflammatory cytokines, such as IL-6 and TNFα, and higher antioxidant capacity [14, 15], improved neutrophils chemotaxis [16], NK cell cytotoxicity and increased T lymphocytes proliferation [17] as well as a stronger post-vaccination response [12, 18]. Previous studies on immunosenescence did not indicate which aspects of age-related immune changes are driven by exercise factors and which may be the consequence of a sedentary lifestyle. Future analyses can improve our understanding of the major features of immunosenescence and the impact of regular physical activity on the immune system in old age [19]. Therefore, the present study was designed to evaluate the relationship between lifestyle exercise and percentage of CD4^+^ and CD8^+^ naïve and memory T lymphocytes as well as CD4/CD8 ratio in active compared to inactive older adults.

## Methods

### Participants

Ninety-nine participants were recruited from the University of the Third Age (U3A), which is an organization encouraging the older adults over 55 years of age to stay active by participating in many educational programmes, including arts, classical studies, discussion classes, computer courses, crafts, drama, film/cinema studies, history, languages, literature, music, sciences, social sciences, and physical activity. The current health status and lifestyle of the participants were controlled by using the health history questionnaire [20]. The age of 60–90 years and the same access to medical healthcare were the inclusion criteria. The exclusion criteria, based on the medical interview, were as follows: acute infectious and oncologic diseases, cardiovascular, neurological and musculoskeletal disturbances and an implemented pacemaker. Moreover, twenty-two older adults withdrew from the project during the study due to high blood pressure above 180/110 mmHg, hospitalization, serious knee injury or a cold. Eventually, the study included fifty-four individuals aged 65–88 years (females *n* = 47, males *n* = 7) who represented the successful ageing according to the definition by Geard et al. [21]. According to the gait speed measurement (6-min walk test), the Åstrand-Ryhming bike test and Community Healthy Activities Model Program for Seniors (CHAMPS) questionnaire, the participants were classified into physically active (*n* = 34) and inactive (*n* = 20) groups (Table 1). All participants were informed of the aim of the study and gave their written consent for participation in the project. The protocol of the study was approved by The Bioethics Commission at Regional Medical Chamber Zielona Gora, Poland (N^o^01/66/2017, N^o^21/103/2018) in accordance with the Helsinki Declaration.

**Table 1:** Anthropometrics and body composition as well as physical activity level (mean ± SD)

	Active *n* = 34	Inactive *n* = 20	Active vs. Inactive *p* level	*η*^2^
**Age** [yr.]	70.2 ± 5.8	73.5 ± 5.4	< 0.05	0.008
**Weight** [kg]	69.8 ± 11.8	67.1 ± 11.3	0.567	0.000
**Height** [cm]	160.3 ± 6.0	159.7 ± 7.2	0.573	0.040
**BMI** [kg/m^2^]	27.1 ± 3.6	26.3 ± 4.1	0.529	0.006
**FM** [kg]	24.1 ± 5.8	22.0 ± 5.9	0.268	0.004
**FM**%	34.3 ± 4.7	32.7 ± 6.2	0.348	0.006
**FFM** [kg]	45.7 ± 7.7	45.1 ± 8.7	0.622	0.012
**SBP** [mmHg]	145.1 ± 19.2	151.3 ± 20.9	0.264	0.017
**DBP** [mmHg]	81.2 ± 11.6	79.2 ± 12.9	0.602	0.004
**6MWT** [m]	527 ± 52	388 ± 59	< 0.001	0.662
**Gate speed** [m/s]	1.5 ± 0.1	1.0 ± 0.1	< 0.001	0.662
**VO**_**2**_**max** [mL/kg/min]	35.8 ± 5.7	32.8 ± 4.0	0.131	0.060

Abbreviations: *BMI* Body Mass Index, *FM* Fat Mass, *FFM* Fat-Free Mass, *SBP* systolic blood pressure, *DBP* diastolic blood pressure, *6MWT* 6-min walking test, *VO*_*2*_*max* maximal oxygen consumption. The measurements in groups are compared by the one-way ANOVA or the Mann-Whitney non-parametric test (if the normality assumption is violated)

### Body composition

Body mass (BM) and body composition fat-free mass (FFM) and fat mass (FM) were evaluated by a bioelectrical impedance method using Tanita Body Composition Analyser MC-980 (Japan) calibrated prior to each test session in accordance with the manufacturer’s guidelines. Duplicate measurements were made standing upright and the average value was included for the final analysis. The recurrence of measurement was 98%. The measurements were taken between 7:00 and 9:00 a.m., before blood sampling in accordance with the procedure previously used by us in older adults [15, 22].

### Functional fitness

The 6-min walking test (6MWT) was accomplished according to technical standards of European Respiratory Society and American Thoracic Society [23]. A marked walkway was laid out in a 50-m rectangular area (dimensions: 20 × 5 m), with cones placed at regular intervals to indicate the distance covered. The aim of the test was to walk as fast and as far as possible within the allotted time (6 min). The total distance walked in the test was recorded and the 6MWT gait speed was then calculated by the following equation: 6MWT gait speed (m/s) = total distance(m)/360 s. The gait speed ranging from 1.3 to 1.8 m/s classified the participants as physically active and the gait speed < 1.3 m/s classified them as inactive according to Middleton et al. [24].

### Cardiorespiratory fitness

The measurement of maximal oxygen consumption (VO_2_max) was executed via the indirect method known as the Åstrand-Ryhming bike test (6-min submaximal exercise test) which depends on the linear relationship between heart rate (HR) and VO_2_ to predict maxVO_2_ and which is approved for both men and women of different ages [25]. Each participant performed a 6-min submaximal exercise test on a cycle ergometer eBike GE Healthcare (Germany). Initially, the study participants rested for 15 min prior to the measurement of their resting HR. According to normative data for submaximal exercise test, the participants who reached the values of VO_2_max > 35 mL/kg/min were classified as active (high activity level) and the remaining ones were determined as inactive (average and low activity level).

### Type and amount of physical activity

The type and weekly amount of physical activity was evaluated by CHAMPS questionnaire [26]. The CHAMPS was originally designed to assess the types and intensity levels of physical activity including lighter (e.g. leisurely walking, water gymnastics, stretching, Tai-Chi) as well as more vigorous activities (e.g. dancing, cycling, swimming). Currently, the CHAMPS also includes a group of items related to a sedentary lifestyle e.g. sitting and chatting with friends.

### Blood sampling

Blood samples were taken from the median cubital vein using S-Monovette-EDTA K_2_ tubes (Sarstedt, Austria) for flow cytometry analysis and morphology and S-Monovette - serum tubes were used for other biochemical markers. Serum samples were left to clot for 45 min before centrifugation and then centrifuged at 3000 g for 10 min. Aliquots of serum were stored at − 80 °C.

### Flow cytometry analysis

**Fig. 1:**
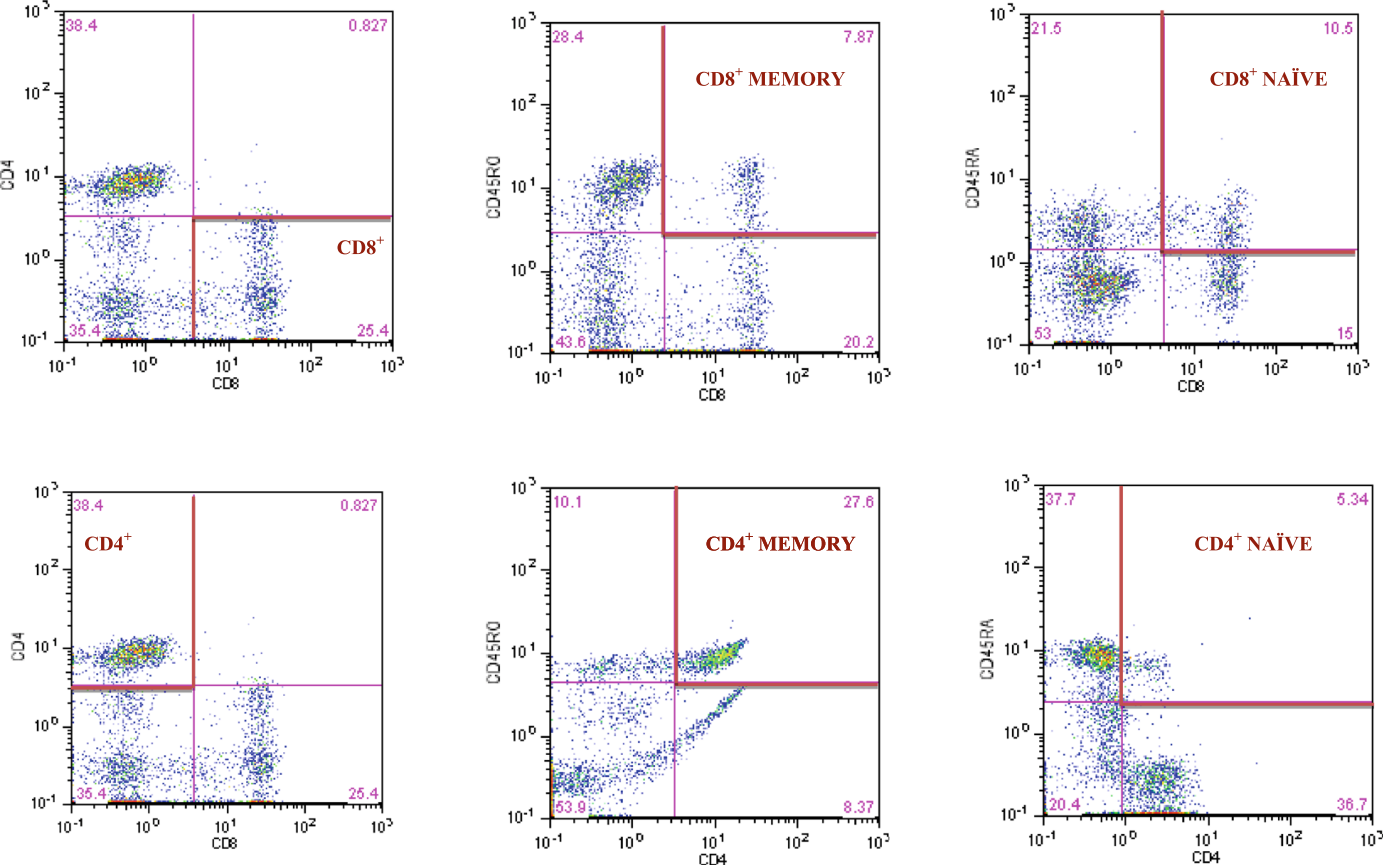
Gating strategy to identify the CD4^+^ and CD8^+^ T lymphocyte and the frequency of CD4^+^ and CD8^+^naïve and memory T lymphocytes

Cytometric analysis was performed using eight-parameter CyFlow Space Sorter flow cytometer by Sysmex Partec (Germany). For the analysis of immune cells, CyLyse kit by Sysmex (Germany) was used. One hundred microliter venous blood was mixed with fluorochrome labelled monoclonal antibodies (CD8 APC, CD4 FITC, CD45 RA Pacific Blue™ CD45RO PE) and incubated for 15 min in the dark at room temperature. After the incubation 100 μL of Leukocyte Fixation Reagent A was added and incubated again in the dark for 10 min. In the last step, 2.5 ml Erythrocytes Lysing Reagent B was added, mixed and incubated in the dark for 20 min and further measurements were made. T helper and cytotoxic lymphocytes were gated by positive surface staining for CD4 and CD8 and were expressed as a percentage of gated lymphocytes. Memory and naïve subpopulations were gated by positive surface staining for CD45RO and CD45RA, respectively. The strategy of gated lymphocytes T was shown in Fig. 1. The ratios of CD4^+^CD45RA^+^ to CD4^+^CD45RO ^+^ and CD8^+^CD45RA^+^ to CD8^+^CD45RO^+^ as prognostic markers of chronic diseases, were calculated according to Hang et al. [27]. The CD4/CD8 ratio was calculated according to McBride and Striker [28] and the reference values were adopted from Strindhall et al. [29]. The ratios ≥1 or ≤ 2.5 are generally considered normal, however, a wide heterogeneity exists because of sex, age, ethnicity, genetics, environmental exposures and infections. The inverted or high CD4/CD8 ratio (< 1 or > 2.5) is regarded as an immune risk phenotype and is associated with immunosenescence and chronic inflammatory diseases [29].

### Haematological variables

Peripheral blood morphology: white blood cell count (WBC), granulocytes (%GRA), lymphocytes (%LYM), red blood cells count (RBC), haemoglobin (HB), haematocrit (HCT), mean corpuscular volume (MCV), mean corpuscular haemoglobin (MCH), mean corpuscular haemoglobin concentration (MCHC), platelets (PLT) were determined by using 3 diff BM HEM3 Biomaxima (Poland).

### Biochemical markers

Total cholesterol (TC), high-density lipoproteins (HDL), low-density lipoproteins (LDL), triglycerides (TG) were determined by using BM200 Biomaxima (Poland). Non-HDL cholesterol was calculated by subtracting HDL from the total cholesterol concentration. CRP was measured using a high sensitivity assay in duplicate by means of a commercial kit from DRG International (USA) with the detection limit of 0.001 mg/L. The intra-assay coefficient of variation (intra-assay CV) for the CRP ELISA kit was 4.44%, and the inter-assay coefficient of variation (inter-assay CV) was 3.28%. The serum glucose was evaluated by using commercially available reagents and a mobile spectrophotometer DP 310 Vario II (Germany).

### Cytomegalovirus (CMV) IgG

The CMV IgG serostatus was determined using a commercial kit from DRG International (USA). The reference values of CMV-seronegativity (IgG CMV^−^) were < 9 DU/mL and CMV-seropositivity (IgG CMV^+^) > 11 DU/mL. The intra-assay CV for the CMV kit was 7.75% and inter-assay CV was 11.45%.

### Statistical analysis

Statistical analyses were performed using the R system, version 3.6.1 [30]. The assumptions for the use of parametric or non-parametric tests were checked using the Shapiro-Wilk and Levene’s tests to evaluate the normality of the distributions and the homogeneity of variances, respectively. The significant differences in mean values between the groups (active vs. inactive) were assessed by the one-way ANOVA. The analysis of covariance (ANCOVA) was used in classification of body composition and functional fitness that might influence the covariate variables such as CMV IgG concentration and T lymphocyte phenotypes. The chi-squared (χ^2^) test was used to compare females and males using categorical data from CHAMPS questionnaire. If the normality and homogeneity assumptions were violated, the Mann-Whitney non-parametric test was used. Additionally, eta-squared (*η*^2^) was used as a measure of effect size which is indicated as having no effect if 0 ≤ *η*^2^ < 0.01, a minimum effect if 0.01 ≤ *η*^2^ < 0.06, a moderate effect if 0.06 ≤ *η*^2^ < 0.14, and a strong effect if *η*^2^ ≥ 0.14 [31, 32]. Spearman’s rank correlation (r_s_ - Spearman rank correlation coefficient) was calculated to describe the relationships between body composition and T lymphocyte count. Statistical significance was set at *p* < 0.05.

## Results

### Body composition

**Fig. 2:**
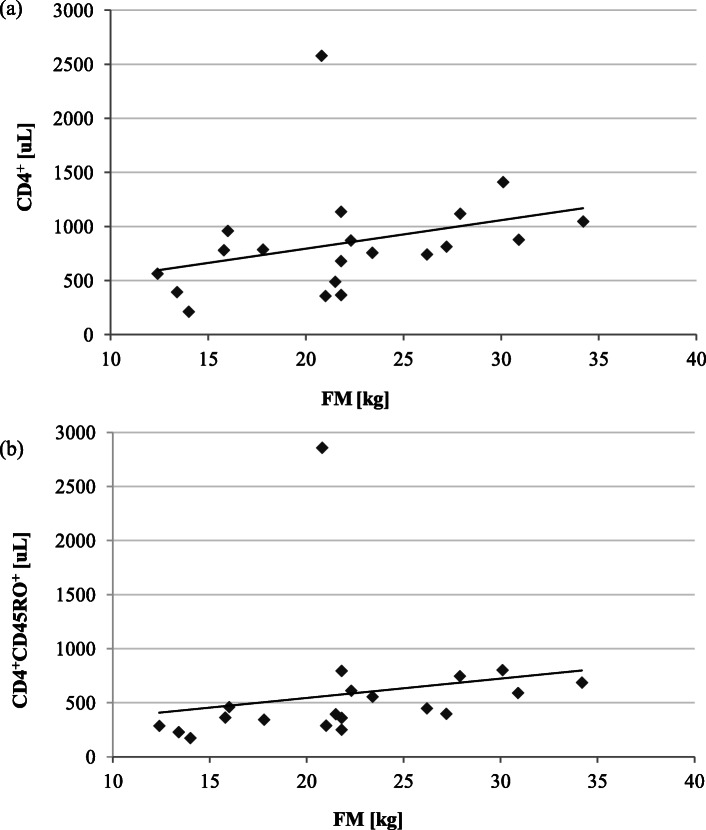
The relationships between fat mass (FM) and CD4^+^ lymphocyte count in the physically inactive group (*n* = 20): **a** FM and CD4^+^ (r_s_ = 0.491, *p* < 0.05), **b ** FM and CD4^+^CD45RO^+^ (r_s_ = 0.636, *p* < 0.01)

The body mass index (BMI) in the physically active older adults ranged from 19.4 to 35.0 kg/m^2^ whereas in the physically non-active older adults their BMI fell within 19.0 to 37.0 kg/m^2^ range (Table 1). Jointly, in both groups, approximately 20% were classified as obese (BMI > 30 kg/m^2^). The BMI value highly correlated with fat mass in active (r_s_ = 0.806, *p* < 0.001) and inactive individuals (r_s_ = 0.783, *p* < 0.001). In the physically inactive group, the high fat mass significantly correlated with CD4^+^ (r_s_ = 0.491, *p* < 0.05) and CD4^+^CD45RO^+^ lymphocytes (r_s_ = 0.636, *p* < 0.01) (Fig. 2). This shows that a low level of everyday activities impairs immunity more considerably in the obese than in the slim older adults.

### Functional and cardiorespiratory fitness

The result of the 6MWT was 36% higher in active than inactive group (Table 1). Above 95% of the study participants achieved a normal gait speed > 1 m/s according to reference values by Middleton et al. [24]. High physical fitness level was confirmed by VO_2_max value which was elevated by 10% in active individuals. Interestingly, the older adults who demonstrated a superior gait speed > 1.3 m/s and VO_2_max > 35 mL/kg/min demonstrated a significantly higher CD4^+^CD45RA^+^T lymphocyte population and a higher CD4^+^CD45RA^+^/CD4^+^CD45RO^+^ ratio compared to the inactive group. In turn, the inactive older adults whose gait speed amounted to 1.0 ± 0.1 m/s, demonstrated VO_2_max < 35 mL/kg/min and had a significantly higher percentage of both CD4^+^CD45RO^+^ and CD8^+^CD45RO^+^T lymphocytes compared to the active individuals. The disproportion in the naïve and memory T lymphocytes emphasises the important role of physical activity in the immunity in the older adults. The value *η*^*2*^ indicated a strong effect of lifestyle exercise on the result of the gait speed test and a moderate effect on VO_2_max value. The most physically active participants, regardless of their gender, indicated Tai-Chi as their favourite type of physical activity (60% of respondents in CHAMPS questionnaire) as opposed to leisurely walking, water gymnastics or Nordic-walking. Tai-Chi was already earlier recommended as an intervention for a wide variety of health problems as it can contribute to reduction of chronic inflammation in the older adults [33].

### Flow cytometry analysis

**Fig. 3:**
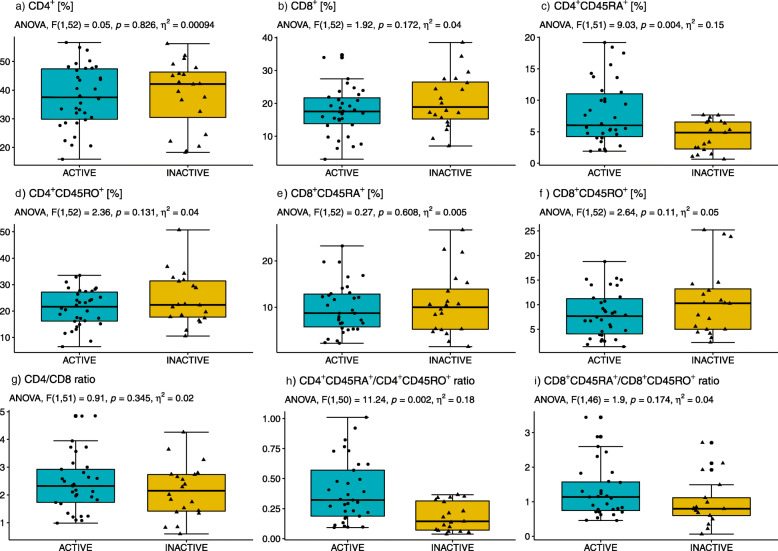
Percentages of total CD4^+^ (**a**) and CD8^+^ T lymphocytes (**b**) and CD4^+^CD45RA^+^ T lymphocytes (**c**) and CD4^+^CD45RO^+^ T lymphocytes (**d**), CD8^+^CD45RA^+^ T lymphocytes (**e**) and CD8^+^CD45RO^+^ T lymphocytes (**f**) as well as the ratios CD4/CD8 (**g**) CD4^+^CD45RA^+^/CD4^+^CD45RO^+^ (**h**) and CD8^+^CD45RA^+^/CD8^+^CD45RO^+^ (**i**) in active compared to inactive older adults

CD4^+^ and CD8^+^ T lymphocytes were analysed within naïve and memory subpopulations in the study groups of active and inactive older adults (Fig. 3, Table 2). The percentage of CD8^+^ T lymphocytes showed a tendency towards low values in physically active compared to inactive older adults participants but the differences did not reach the level of significance (Fig. 3b). The active study group of older adults was found to exhibit a decreased percentage of both CD4^+^CD45RO^+^ and CD8^+^CD45RO^+^ T lymphocytes (Fig. 3d  and f). However, the percentage of CD4^+^CD45RA^+^ (Fig. 3c) and the CD4^+^CD45RA^+^/ CD4^+^CD45RO^+^ ratio (Fig. 3h) were significantly higher in the physically active group compared to the inactive one. There were no statistically significant differences between males and females in either of the groups (active vs. inactive). The value *η*^*2*^ indicated a strong impact of lifestyle exercise on the CD4^+^CD45RA^+^ T lymphocytes as well as on the ratio of CD4^+^CD45RA^+^/CD4^+^CD45RO^+^. The CD4/CD8 ratio was observed to be higher in the active than inactive individuals (Fig. 3g). 55.9% of the physically active older adults demonstrated CD4/CD8 ratio ≥ 1 or ≤ 2.5, 41.2% of them exhibited the CD4/CD8 ratio > 2.5, and the ratio < 1 was recorded only in 2.9%. The CD4/CD8 ratio ≥ 1 or ≤ 2.5 was detected in approximately 45% of the inactive older adults, whereas 40% of them exhibited the CD4/CD8 ratio > 2.5, and only 15% showed the CD4/CD8 ratio < 1. This shows that inactive lifestyle especially shifts the values of CD4/CD8 ratio < 1. The changes in CD4/CD8 ratio also depend on BMI (normal, overweight and obesity) in both active and inactive older adults. The majority of active older adults participants, regardless of body mass, had a CD4/CD8 value within the reference range and only 14% of active adults with obesity had an inverted CD4/CD8. In turn, the majority of the inactive older adults, regardless of their body mass, manifested CD4/CD8 ratio > 2.5 and approximately 30% of older inactive adults with overweight had CD4/CD8 < 1 (Table 3). The results clearly indicate that a low level of lifestyle exercise changes the CD4/CD8 ratio included in IRP which, in turn, is related to immunosenescence and chronic inflammatory diseases [29].

**Table 2:** Changes in T lymphocytes absolute count (mean ± SD)

T lymphocytes absolute count [μL]	Active *n* = 34	Inactive *n* = 20	Active vs. Inactive *p* level	*η*^2^
**CD4**^**+**^	880.8 ± 363.7	846.3 ± 506.4	0.210	0.031
**CD8**^**+**^	414.6 ± 225.3	446.7 ± 262.7	0.560	0.007
**CD4**^**+**^**CD45RA**^**+**^	181.9 ± 113.5	108.0 ± 88.3	< 0.01	0.192
**CD4**^**+**^**CD45RO**^**+**^	494.8 ± 225.8	579.8 ± 568.8	0.784	0.002
**CD8**^**+**^**CD45RA**^**+**^	233.1 ± 149.4	230.1 ± 164.5	0.920	0.001
**CD8**^**+**^**CD45RO**^**+**^	187.2 ± 115.9	252.7 ± 299.5	0.649	0.004
**CD4/CD8**	2.5 ± 1.1	2.1 ± 1.0	0.345	0.018
**CD4**^**+**^**CD45RA**^**+**^**/CD4**^**+**^**CD45RO**^**+**^	0.4 ± 0.3	0.3 ± 0.3	< 0.001	0.179
**CD8**^**+**^**CD45RA**^**+**^**/CD8**^**+**^**CD45RO**^**+**^	1.6 ± 1.1	1.6 ± 1.7	0.132	0.040

The measurements in groups are compared by the one-way ANOVA or the Mann-Whitney non-parametric test (if the normality assumption is violated)

**Table 3:** Changes in the CD4/CD8 ratio depending on body mass index (BMI)

	Active *n* = 34			Inactive *n* = 20		
CD4/CD8 ratio	< 1	≥ 1 or ≤ 2.5	> 2.5	< 1	≥ 1 or ≤ 2.5	> 2.5
**Normal weight** BMI 18.5–24.9 kg/m^2^	0%	60%	40%	10%	60%	30%
**Overweight** BMI 25.0–29.9 kg/m^2^	0%	53%	47%	28.6%	28.6%	42.8%
**Obesity** BMI ≥30 kg/m^2^	14.3%	57.1%	28.6%	0%	33.3%	66.7%

### CMV IgG status and immune cells (Table 4)

88% of the active older adults participants were CMV-seropositive and the mean value of IgG CMV^+^ was 98.1 *±* 21.21 DU/mL and only 12% of the active participants were CMV-seronegative. The results obtained for the inactive older adults showed that 100% of them were CMV-seropositive and the average value reached 92.3 *±* 22.8 DU/mL. In the physically inactive IgG CMV^+^ participants, a decreasing trend of the CD4/CD8 ratio was observed unlike in the physically active individuals with IgG CMV^+^, however, both values ​​of the CD4/CD8 ratio fell within the reference range. The conducted analyses revealed statistically significant differences in the absolute CD4^+^CD45RA^+^ T lymphocytes count as well as the CD4^+^CD45RA^+^/CD4^+^CD45RO^+^ ratio between active CMV-seropositive compared to inactive CMV-seropositive participants. T lymphocyte absolute counts did not differ between CMV-seronegative and CMV-seropositive groups, which can be ascribed to a small size of the CMV-seronegative group. The obtained results suggest that regular physical activity has a positive effect on the immune system, especially in CD4^+^CD45RA^+^ T lymphocyte count, regardless of CMV-seropositivity.

**Table 4:** Distribution of T lymphocytes phenotypes in relation to CMV serostatus (mean ± SD)

T lymphocytes absolute count [μL]	Active IgG CMV^**+**^ *n* = 30	Active IgG CMV^**−**^ *n* = 4	*p* level	*η*^2^	Inactive IgG CMV^**+**^ *n* = 20	Active vs. Inactive *p* level	*η*^2^
**CD4**^**+**^	922.4 ± 349.6	569.0 ± 356.0	0.067	0.101	846.3 ± 506.4	0.258	0.008
**CD8**^**+**^	434.5 ± 226.9	265.2 ± 164.8	0.198	0.060	446.7 ± 262.7	0.992	0.001
**CD4**^**+**^**CD45RA**^**+**^	189.0 ± 112.8	128.9 ± 120.8	0.337	0.030	108.0 ± 88.3	< 0.01	0.132
**CD4**^**+**^**CD45RO**^**+**^	518.2 ± 219.5	319.5 ± 221.0	0.099	0.083	579.8 ± 568.8	0.589	0.006
**CD8**^**+**^**CD45RA**^**+**^	247.6 ± 149.7	123.8 ± 103.9	0.118	0.074	230.1 ± 164.5	0.536	0.003
**CD8**^**+**^**CD45RO**^**+**^	192.8 ± 114.3	145.2 ± 137.1	0.453	0.018	252.7 ± 299.5	0.977	0.020
**CD4/CD8**	2.5 ± 1.0	2.8 ± 1.8	0.541	0.012	2.1 ± 1.0	0.294	0.023
**CD4**^**+**^**CD45RA**^**+**^**/CD4**^**+**^**CD45RO**^**+**^	0.4 ± 0.3	0.4 ± 0.2	0.777	0.000	0.3 ± 0.3	< 0.001	0.061
**CD8**^**+**^**CD45RA**^**+**^**/CD8**^**+**^**CD45RO**^**+**^	1.6 ± 1.2	1.0 ± 0.3	0.393	0.038	1.6 ± 1.7	0.350	0.000

Abbreviations: *IgG CMV*^*−*^ cytomegalovirus seronegativity, *IgG CMV*^***+***^ cytomegalovirus seropositivity. The comparison between active and inactive group concerns CMV seropositive participants. The measurements in groups are compared by the one-way ANOVA or the Mann-Whitney non-parametric test (if the normality assumption is violated)

### Haematological variables (Table 5)

The immune cell numbers (lymphocytes, %LYM, %GRA, %HCT, MCV, MCHC, MCH) were found to be at similar levels in all participants. However, there were significant differences between PLT in the active compared to inactive individuals. Moreover, an increasing trend in HB, RBC and HCT was observed in the physically active group. The value η^2^ indicated a moderate influence of lifestyle exercise on HB level, which suggests that a protective effect of physical activity is also exerted on hematopoietic processes in the older adults. There were no statistically significant differences in HB, RBC and HCT between males and females in both groups (active vs. inactive).

**Table 5:** Haematological variables (mean ± SD)

	Reference values	Active *n* = 34	Inactive *n* = 20	Active vs. Inactive *p* level	*η*^2^
**WBC** [10^3^/μL]	5.0–11.6	6.7 ± 2.1	6.5 ± 2.0	0.740	0.055
**Lymphocytes** [10^3^/μL]	1.3–4.0	2.3 ± 0.6	2.2 ± 0.9	0.119	0.002
**Granulocytes** [10^3^/μL]	2.4–7.6	3.9 ± 1.6	3.8 ± 1.4	0.851	0.076
**LYM** %	19.1–48.5	35.5 ± 7.6	33.7 ± 9.0	0.486	0.003
**GRA** %	43.6–73.4	57.2 ± 9.2	57.2 ± 9.0	0.981	0.000
**RBC** [10^3^/μL]	F 4.0–5.5 M 4.5–6.6	4.8 ± 0.3	4.7 ± 0.3	0.209	0.054
**HB** [g/dL]	F 12.5–16.0 M 13.5–18.0	13.9 ± 0.7	13.7 ± 0.8	0.513	0.108
**HCT** [%]	F 37–47 M 40.0–51.0	39.5 ± 2.0	38.6 ± 2.4	0.201	0.031
**MCV** [fL]	F 80–95 M 80–97	81.9 ± 3.0	81.7 ± 2.6	0.950	0.048
**MCH** [pg]	F 27.0–32.0 M 26.0–32.0	28.7 ± 1.3	29.0 ± 1.1	0.395	0.054
**MCHC** [g/dL]	F 32.0–36.0 M 31.0–36.0	35.1 ± 0.8	35.5 ± 0.6	0.330	0.018
**PLT** [10^3^/μL]	150–400	270 ± 60	236 ± 39	< 0.01	0.031

Abbreviations: *WBC* white blood cells, *LYM* lymphocytes, *GRA* granulocytes, *RBC* red blood cells, *HB* haemoglobin, *HCT* haematocrit, *MCV* mean corpuscular volume, *MCH* mean cells haemoglobin, *MCHC* corpuscular/cellular haemoglobin concentration, *PLT* platelets, *F* female, *M* male. The measurements in groups are compared by the one-way ANOVA or the Mann-Whitney non-parametric test (if the normality assumption is violated)

### Biochemical markers (Table 6)

The glucose and TG concentrations did not exceed the limits of the reference values in most study participants. TC concentration was found to fall within the range of 157 to 372 mg/dL in the physically active participants whereas in the inactive group it amounted to the values from 162 to 394 mg/dL. High TC concentration > 200 mg/dL was observed in approximately 87% of the study participants and only 5 participants took lipid-modifying drugs. Similar observations were made with regard to the changes in LDL, HDL and non-HDL concentrations. CRP level tended to reach higher values in the active than inactive participants but remained within the reference range. However, a lower CRP concentration (2.62 ± 1.88 mg/L) was found in the older adults who demonstrated a superior gait speed ≥1.4 m/s when compared to the other active participants (4.19 ± 2.75 mg/L). CRP concentration was inversely correlated with the results of the 6MWT or gait speed test (r = − 0.350, *p* < 0.05) in the active older adults, which clearly indicates that lifestyle exercise diminishes the systemic inflammatory response.

**Table 6:** Lipoprotein-lipid profile, glucose and C-reactive protein (mean ± SD)

	Reference values	Active *n* = 34	Inactive *n* = 20	Active vs. Inactive *p* level	*η*^2^
**Glucose** [mg/dL]	60–115	98.6 ± 17.1	97.6 ± 20.3	0.452	0.000
**TG** [mg/dL]	< 150	90.2 ± 25.7	83.7 ± 26.0	0.375	0.000
**TC** [mg/dL]	< 200	262.4 ± 52.6	251.3 ± 59.1	0.534	0.013
**LDL** [mg/dL]	< 130	147.1 ± 40.3	149.1 ± 50.8	0.889	0.000
**HDL** [mg/dL]	desirable > 60	82.2 ± 16.0	81.1 ± 11.8	0.603	0.023
**non-HDL** [mg/dL]	< 130	180.2 ± 54.7	170.2 ± 65.5	0.599	0.047
**CRP** [mg/L]	0.068–8.2	3.2 ± 2.5	2.8 ± 3.1	0.121	0.004

Abbreviations: *TG* triglycerides, *TC* total cholesterol, *LDL* low density lipoprotein, *HDL* high density lipoprotein, *CRP* C-reactive protein. The measurements in groups are compared by the one-way ANOVA or the Mann-Whitney non-parametric test (if the normality assumption is violated)

## Discussion

Regular physical activity has a profound effect on normal functioning of the immune system. For decades it has been accepted that prolonged periods of high-intensity exercise could depress immunity [34]. However, current evidence from epidemiological studies shows that leading a physically active lifestyle is likely to be beneficial rather than harmful to the immune function [35]. Exercise-induced improvements in immunity can be related to reduction in inflammation, maintenance of thymic mass, changes in the composition of memory and naïve T lymphocytes or enhanced immunosurveillance. Indeed, physical activity is a powerful intervention that has a great potential to improve immune and health outcomes in the older adults, the obese, and patients with cancer and chronic viral infections [10, 34]. The benefits of regular physical activity undertaken by the older adults are much less documented than the effects of regular physical activity on the immune system in young individuals [36]. Randomized prospective trials were conducted to explain the effect of aerobic exercise on the immune system of the older adults, where Nieman et al. [37] found that a 3-month moderate aerobic exercise programme did not cause a significant increase in T lymphocyte mitogenesis. On the other hand, the effect of resistance training on the immune function has also been poorly investigated and most researchers agreed that 8–12-week training had minimal effects on innate or acquired immunity in older adults [38]. It is well known that physical activity does not only exert anti-inflammatory effects but also positively affects the metabolic health in the older adults. The results of CHAMPS questionnaire in our study confirmed the activity of our recruited participants in everyday life, where older adults who were physically active were much more likely to lead a more active life by being engaged in Nordic walking, swimming, intensive walks, and Tai-Chi, than inactive older adults participants. In our study, the active older adults covered a much longer distance in the 6MWT compared to the inactive older adults. In addition, the active older adults demonstrated the gait speed of 1.5 ± 0.1 m/s that was significantly higher in comparison to the non-active group whose gait speed amounted to 1.0 ± 0.1 m/s, which can be indicative of better functional fitness of the active older adults. It is believed that a low intensity of everyday activity and a sedentary lifestyle constitute an independent risk of diseases known as „sedentariness”. A longitudinal Canadian study of 17,013 people aged over 12 years demonstrated that those who were inactive for a longer time span were up to 50% more likely to die prematurely in comparison to individuals who spent shorter periods of time in a sitting position [39].

In addition to beneficial effects on the immune system, physical activity throughout lifetime also favourably affects well-being and facilitates daily functioning in society [40]. Good functional status of the older adults participants could be related to their participation in various physical and health education programmes at the University of the Third Age. In recent years the effects of regular physical activity on T lymphocytes have attracted a considerable interest and plenty of evidence showed the lifestyle exercise may lead to rejuvenation of the immune system and may exert a positive effect on thymic output. The active older adults in our study were observed to have a statistically significantly increased percentage of blood CD4^+^CD45RA^+^ T lymphocytes in comparison to the inactive older adults. According to Weyh et al. [41], this may be associated with elevated IL-15 levels that affect the immune homeostasis which is caused by the induction of a better survival rate of naïve T lymphocytes. Duggal et al. [19] noticed some reduction in systemic inflammation in amateur cyclists (mean age: 55–75 years) engaged in high levels of physical activity for most of their adult life. Spielmann et al. [42] also found that males aged 18–61 years, with high VO_2_max level, demonstrated an increased number of CD8^+^ naïve T lymphocytes.

Attempts to determine the relationships between CMV-seropositivity and changes in the count of T lymphocytes have been undertaken by scientists for many years. The results of the research carried out due to the health condition of the examined patients, genetic background and/or many others factors in highly diverse human populations are varied. Most researchers agree that CMV infection at least accelerates the age-related decrease in the number of naïve T lymphocytes and the increase in memory T lymphocytes [43]. In our study, we showed that, regardless of CMV-seropositivity, in the physically active older adults there was an increase in the count of CD4^+^CD45RA^+^ T lymphocytes as well as in the CD4^+^CD45RA^+^/CD4^+^CD45RO^+^ ratio compared to the inactive CMV-seropositive older adults. This emphasises the beneficial effect which the activity of older adults exerts on their immune system functioning. Latent infection in people with normal immunity frequently shows no symptoms, but it could be dangerous for immune compromised ones. This is associated with CD4/CD8 < 1 identified in immune-risk individuals, which induces a high risk of mortality due to weaker immune response [44]. In our study, the inactive older adults CMV-seropositive individuals showed a lower CD4/CD8 ratio compared to the active older adults CMV-seropositive adults. Interestingly, older active CMV-seronegative adults obtained the CD4/CD8 ratio of 2.8 ± 1.5 which is higher than that observed in the active older CMV-seropositive adults (2.5 ± 1.0) as well as in the inactive CMV-seropositive individuals (2.1 ± 0.9). Nevertheless, due to the limited number of IgG CMV-seronegative ^−^ individuals we were not able to draw unequivocal conclusions and the research must be continued.

During the past decade, three prospective cohort studies with the participation of Swedes, Dutch and Belgians were performed to assess the IRP in the older adults defined by the CD4/CD8 ratio [45]. The inconsistencies may be ascribed to a large number of factors, including gender, age, nutrition, amount of physical activity or fat content, which can all affect the ratio, and also to the fact that the values ≥1 or ≤ 2.5 are commonly used as the reference values in healthy individuals [28]. The CD4/CD8 ratio can also be a useful marker to determine the body response to lifestyle exercise. Researchers have not yet unequivocally established whether the CD4/CD8 ratio increases or decreases with age. Neither are they unanimous as to whether the rise or the decline in the ratio is more favourable to maintain the longevity of the older adults. The CD4/CD8 ratio was found to increase with age in OCTO/NONA surviving participants over 100 years of age [29]**.** On the other hand, the analysis by Vasson et al. [46] showed a decreasing trend of the CD4/CD8 with age in Spanish and French population. In our study, we searched for the answer whether lifestyle exercise had an effect on the CD4/CD8 ratio. Interestingly, the CD4/CD8 ratio was found to fall within the range of the reference values in 55.9% of the group of older active participants. Our study group of active older adults was classified as representing healthy ageing. The frequency of the CD4/CD8 ratio is also contingent on the body fat content and in all our study participants (both active and inactive), high fat content shifted the CD4/CD8 ratio < 1.

Ageing is commonly accompanied by abdominal obesity, which is often associated with health problems and with an increased risk of infections. Adipose tissue acts as a ‘link’ between nutrition, metabolism and the proper functioning of the immune system in healthy individuals [47]. Furthermore, ageing does not only induce adiposity but it also causes changes in body composition, such as the loss of muscle mass, muscle fat infiltration or bone loss [48], and the ensuing decline in physical performance adversely affects the quality of life [49]. Changes in body composition are a significant risk factor for developing impaired physical performance, however, there is little evidence that the FFM and FM indices are associated with physical disability in older adults, women and men alike [48].

It is worth mentioning that visceral adipose tissue contains the major immune cells which play a critical role in immune-metabolic homeostasis. The changes in adipose tissue T lymphocytes in obesity have been well documented in mice and humans [47]. In obese individuals the percentage of T lymphocytes of both CD4 and CD8 cells in adipose tissue is on the increase, secreting pro-inflammatory cytokines such as: IFN-γ (Th1) and IL-17 (Th17). The increased proportion of inflammatory CD4^+^ T lymphocytes in obese individuals results in a decrease in the number of T_reg_ [50].

To date, only a limited number of studies have analysed the composition of the peripheral blood of the immune system in obesity. In our study, a positive correlation between CD4^+^ T lymphocyte count and FM was observed in the inactive older adults. This finding suggests that the CD4^+^ T lymphocyte pool is on the rise along with the increase in the body fat. The results are consistent with the ones obtained by van der Weerd et al. [51], who reported an increase in the number of CD4^+^ T lymphocytes in obese people. Womack et al. [52] analysed both CD4^+^ and CD8^+^ T lymphocytes in African women and they also recorded an increased T lymphocyte pool in the obese study participants. However, Tanaka et al. [53] observed a decrease in the T lymphocyte (CD4^+^ and CD8^+^) population in the peripheral blood of 34 obese individuals as compared to 50 non-obese participants. Contrastingly, O’Rourke et al. [54] demonstrated an elevated number of CD4^+^ T lymphocytes and a reduced number of CD8^+^ T lymphocytes in morbidly obese women compared to healthy, normal-weight controls. A potential mechanism related to such divergent results is a consequence of gender and age diversity of the study population as well as a small sample size. Therefore, the relationship between body mass and the CD4^+^ T lymphocyte pool invites additional consideration. What calls for further investigation is also the level of leptin, a hormone that is involved in the development of thymus gland especially in the differentiation of thymocytes from double positive cells to single positive CD4^+^ and CD8^+^ T lymphocytes [55]. One of potential mechanisms of leptin has been proven to increase the production of naïve T lymphocytes (CD4^+^CD45RA^+^CD45RO^−^) and to inhibit the proliferation of memory T lymphocytes (CD4^+^CD45RO^+^CD45RA^−^) [56]. It is still not entirely clear how obesity-induced metabolic dysfunction affects and changes the imbalance in the naïve and memory T lymphocytes population. Naïve T lymphocytes remain at rest and their activation is contingent on the energy demand for phosphorylation. After activation, the metabolic signature changes to provide support for increased glycolysis to fulfil cellular energy requirements [57]. According to Pearce et al. [58], the development and survival of memory T lymphocytes is reliant on fatty-acid oxidation (FAO). In our research we also demonstrated a positive correlation between FM and CD4^+^CD45RO^+^ T lymphocyte count. Yang et al. [59, 60] observed that obesity accelerated the age-related reduction of T-lymphocyte receptor, which was connected with reduced thymopoiesis. Obesity was observed to lead to reduction in peripheral naïve T lymphocytes with increased frequency of effector-memory T lymphocytes. The precise role of leptin and the association between FM and immune cells, T lymphocytes in particular, in older individuals shall be analysed in further investigation in our cohort.

Appropriate strategies to counteract immunosenescence should be implemented and one of them should take into account the beneficial effect of lifestyle exercise on subpopulations of the immune system [61]. To date, numerous studies have confirmed a favourable impact of physical activity on the immune system including an enhanced proliferative capacity of T lymphocytes and magnified cytotoxic activity of NK cells. The extent of exercise-induced changes to the immune system in the older adults is quite diverse and depends on the type of physical activity as well as its volume and intensity. Future analyses will allow us to understand the role which physical activity sustained throughout life plays on prevention of immunosenescence and lifestyle diseases [62].

## Conclusions

In this study we demonstrated that major features of immunosenescence were driven by lifestyle exercise. Physical activity sustained throughout life enhances the immune system by increasing the percentage of naïve T lymphocyte population and by reversing the CD4^+^CD45RA^+^/CD4^+^CD45RO^+^ ratio. Interestingly, the beneficial changes in the CD4/CD8 ratio were observed at the values of ≥1 or ≤ 2.5 in ~ 50% of physically active older adults regardless of their body weight, which classified them as successfully ageing older adults, contrary to the inactive and obese older adults who demonstrated the CD4/CD8 ratio < 1.

### Limitations

The limitations of the study include a relatively small number of participants especially male individuals. Lack of information on the exposure to pathogens throughout life of study participants may also have affected the disproportions in the populations of the analysed cells. Moreover, some lifestyle factors e.g. diet, were not taken into account in our analysis, which should also be acknowledged as the limitation of the study.

## Data Availability

The datasets used and/or analysed during the current study are available from the corresponding author on reasonable request.

## Abbreviations

BMI: Body mass index
CHAMPS: Community Healthy Activities Model Program for Seniors
CMV: Cytomegalovirus
CRP: C-reactive protein
DBP: Diastolic blood pressure
FAO: Fatty-acid oxidation
FFM: Fat-free Mass
FM: Fat mass
GRA: Granulocytes
HB: Haemoglobin
HCT: Haematocrit
HDL: High density lipoprotein
HIV: Human immunodeficiency virus
HR: Heart rate
IRP: Immune risk profile
LDL: Low density lipoprotein
LYM: Lymphocytes
MCH: Mean corpuscular haemoglobin
MCHC: Mean corpuscular haemoglobin concentration
MCV: Mean corpuscular volume
NK: Natural killer
PLT: Platelets
RBC: Red blood cells count
SBP: Systolic blood pressure
TC: Total cholesterol
TCR: T cell receptor
TG: Triglycerides
U3A: University of the Third Age
VO2max: Maximal oxygen consumption
WBC: White blood cells count
6MWT: 6-min walking test
